# Fatigue life analysis for 6061-T6 aluminum alloy based on surface roughness

**DOI:** 10.1371/journal.pone.0252772

**Published:** 2021-06-30

**Authors:** Yali Yang, Hao Chen, Wang Feng, Sha Xu, Yongfang Li, Ruoping Zhang

**Affiliations:** School of Mechanical and Automotive Engineering, Shanghai University of Engineering Science, Shanghai, 201620, China; China University of Mining and Technology, CHINA

## Abstract

Surface condition is one of the dominant factors affecting fatigue life. Considering the complexity of surface condition, a relatively efficient and economic approach based on surface reconstruction and interpolation method was proposed. The effect of surface roughness on the fatigue life of 6061-T6 aluminum alloy is studied to analyze the fatigue life by surface roughness parameters. Surface topography was simplified into a series of elliptic micro notches, and empirical formula for stress concentration factor is established based on simulation work. Then the extraction method of surface curve is proposed to effectively represent the real surface roughness through 3D model reconstruction. Experiment of surface roughness verified the correctness of the model. The relationship between surface roughness and fatigue life is established and the calculated value of the fatigue life is compared with the test results. The maximum error is 15.65%, indicating that the formula established is reasonable and effective.

## 1 Introduction

As fatigue cracks generally initiate at surface, surface condition of component has been one of the dominant factors for fatigue life [1–3]. Fatigue damage on component surface typically develops due to the surface integrity resulting from manufacturing and the presence of stress concentrations originating from surface topography. Fatigue strength of engineering components increases with the decrease of surface roughness [4,5].

As crack can initiate easily from a rougher surface, increasing value of surface roughness is potentially resulting in reduced service life [6,7]. Chen et al [8] pointed out that surface roughness was a controlling factor of fatigue performance, by comparison between machined samples and net-shape samples. Arola et al [9] proposed Arola-Ramulu model based on surface roughness and radius of curvature at the bottom of the notch, the complex surface morphology was simplified into an ideal sinusoidal micro-notch, and the maximum error between calculation and experiment was only 2%. Andrews et al [10] proposed a relationship between stress concentration factor and micro-notch parameters by simplifying surface roughness into general semi-elliptical notches. Zhang et al and Liao et al [11,12] established quantitative relationship between surface roughness and surface stress concentration factor by a semi-elliptical micro-notch characterization model, and determined its effect on fatigue life. However, the stress concentration factor *K*_*t*_ in those studies was not verified by measured surface topography. Several investigations have been conducted by incorporating surface topography measurement. Ås et al [13], Suraratchai et al [14] and Li et al [15] utilized finite element method to calculate the stress concentration factor of 2D profiles from the measured 3D surface topography, and predicted the fatigue life based on fatigue test. Ås et al [16] provided a geometric modeling analysis of the measured 3D surface topography to calculate the stress concentration factor. Dai et al [17] investigated the effect of surface roughness on the fatigue behavior of 2024-T3 aluminum alloy under high and low cyclicloading. Vayssette et al [18] conducted numerical analysis by importing surface scans from profilometry into finite element model, and reported that fatigue strength increase with the decrease of surface roughness.

In this paper, fatigue damage had been studied based on the analysis of surface roughness. Surface topography was simplified into a series of elliptic micro notches, and the influence of surface micro-notch characteristics on the stress concentration factor had been studied. The relationship between surface roughness and stress concentration factor was established. The specimens with real morphology of rough surface were processed and the reconstruction of 3D models were realized in different scales of roughness. Then the fatigue damage was analysed through simulation of models with surface roughness. At last, the relationship between fatigue life and stress concentration factor was established by empirical formula,and this work wasverified by fatigue experiment.

## 2 Material and methods

### 2.1 Specimen

**Fig 1:**
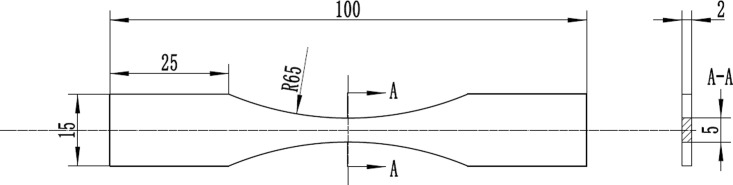
Specimen dimension.

The specimen is fabricated from 6061-T6 aluminum alloy, and the dimension is shown in Fig 1.

### 2.2 FE model with surface micro-notch

**Fig 2:**
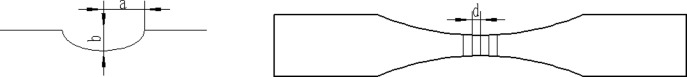
Geometry and dimension of surface notches.

Surface roughness can be considered as a series of microscopic gaps. Assuming that the shape of surface indentation is elliptical, a 3D finite element model of specimen with lateral indentation is established. Fig 2 is the topography and dimensions of the elliptical surface indentations. The depth of notch *b* is varied from 1 to 12μm, the ratio of width to depth of notch *a*/*b* is from 2 to 10, and the distance *d* between two adjacent notches on the surface fluctuates is between 2 to 10 times the width *a* [11].

**Fig 3:**
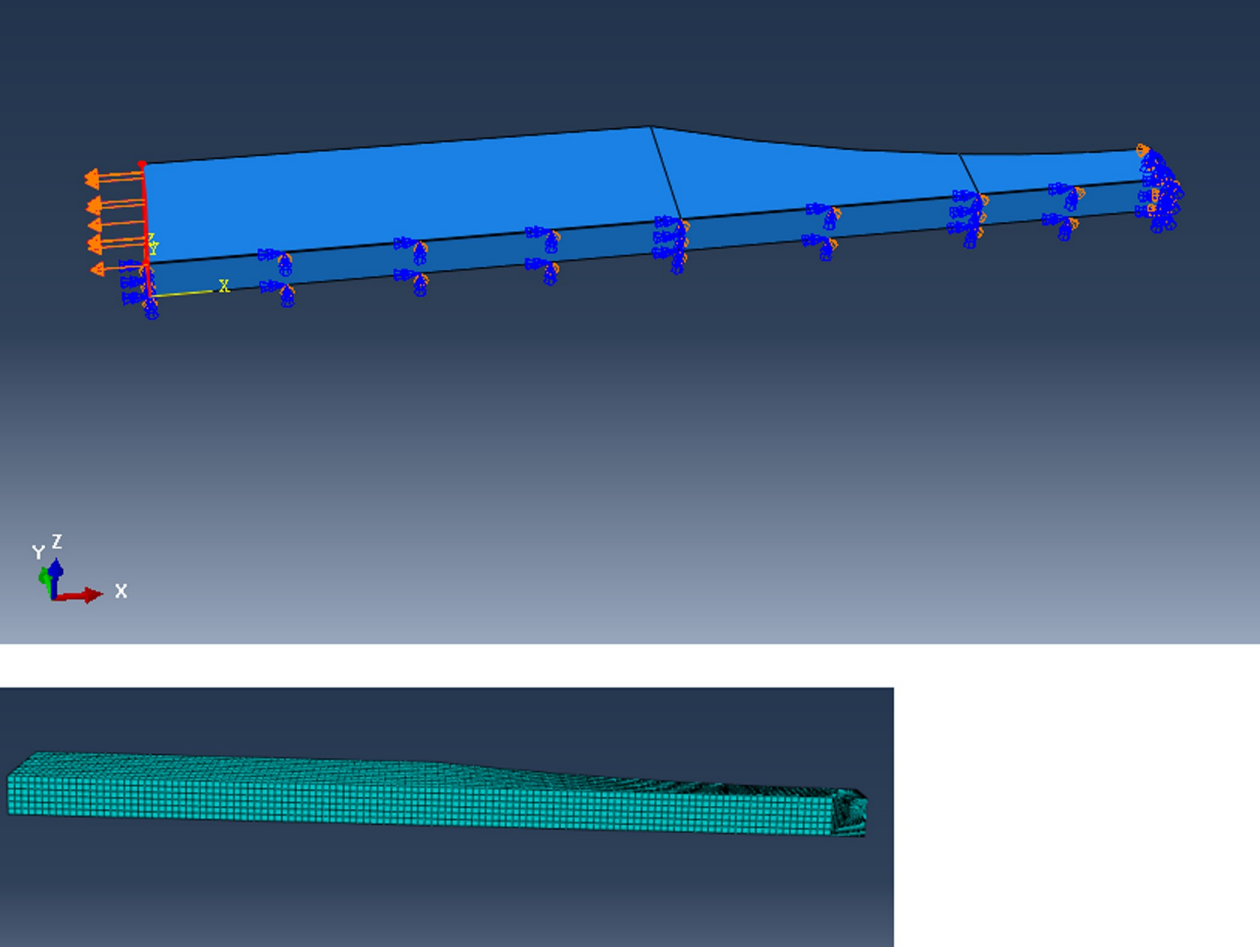
The model with surface roughness in ABAQUS.

A quarter model is built with a fixed constraint applied to one end, and a uniform tensile load P = 103 MPa is applied to another end (Fig 3A). Elliptical notches are inserted with geometry parameters by script files of Abaqus/Python. The material is considered as linear elastic homogeneous isotropic aluminum, *E* = 70000 *MPa*, *v* = 0.33 The type of finite element model is a collapsed element side, and duplicate nodes configure the mid-side node parameter to its 1/4 position to simulate the singularity of displacement of the crack tip region, as shown in Fig 3B.

Surface morphology is characterized by continuous adjacent gaps, and the empirical relationship between micro-notch parameters and stress concentration factor is established by data fitting. Surface roughness parameters are selected to convert the notch parameters into the surface roughness. The relation between surface roughness and stress concentration factor is obtained accordingly.

### 2.3 Surface roughness experiment

**Fig 4:**
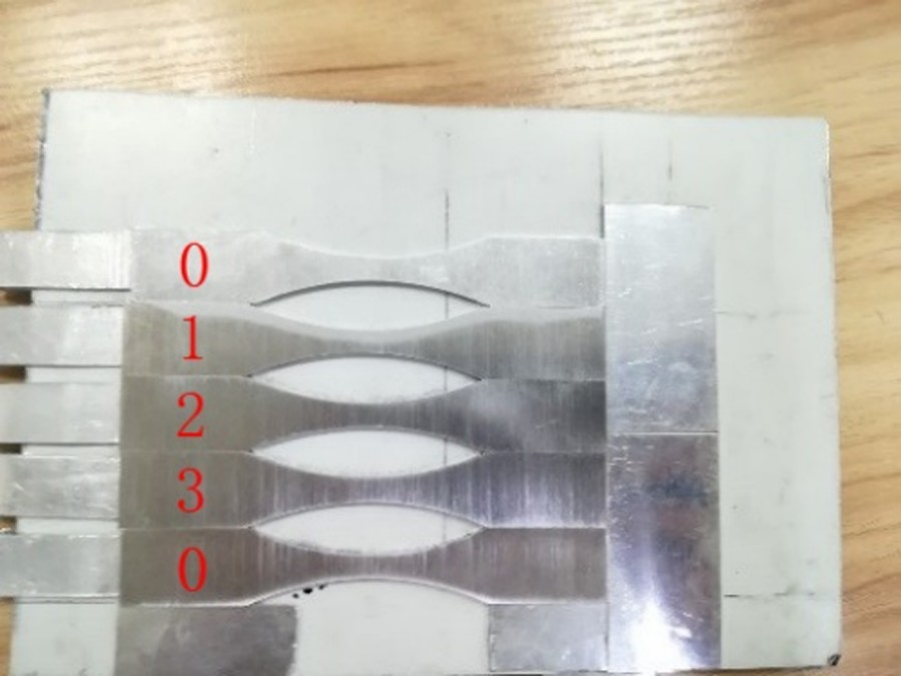
Fixed mode of specimens.

**Fig 5:**
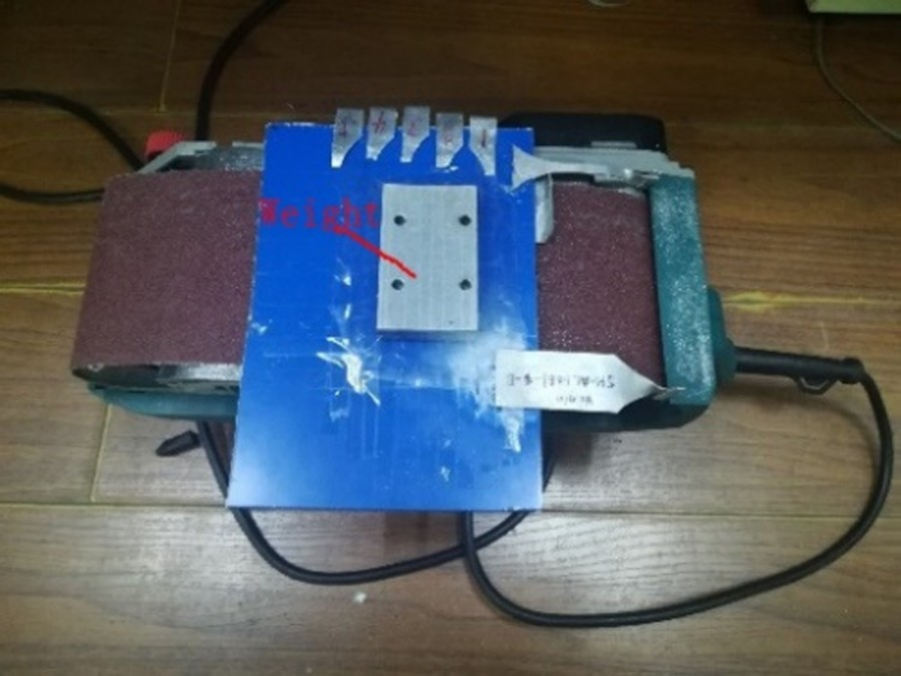
Grinding of specimen.

To obtain real surface roughness data for calculation and verification, four different levels of surface roughness are included by 80, 120, 240, and 400 grit sandpapers with longitudinal sanding, with 3 replications for each level. The test pieces are fixed to fit the specimen closely with the baseboard, as shown in Fig 4. The emery paper is rotated on the roller running 40s at a speed of 210m/min, with applied load on the middle section of the specimen, as shown in Fig 5. Surface roughness is measured by contact profilometry with SJ-210 roughness meter.

### 2.4 Acquisition and reconstruction of surface feature

In order to verify the rationality of the simplified model and the established prediction formula of fatigue life based on surface condition, finite element analysis is conducted based on the reconstruction of real surface morphology of loaded specimens.

The real surface roughness is obtained based on image processing method. First, surface morphology image is obtained by BX53M microscope with image stitching and calibration. The maximum dent depth *R*_*z*_ can be obtained. Then, the edge curve is extracted from images to obtain the height information of the surface topography. The boundary curve of the image is extracted by MATLAB, and the surface roughness curve of each specimen can be obtained. Finally, the surface topography data points after interpolation are imported into 3D modeling software through point cloud technology to generate curve [15].

### 2.5 Finite element analysis of model with surface topography

**Fig 6:**
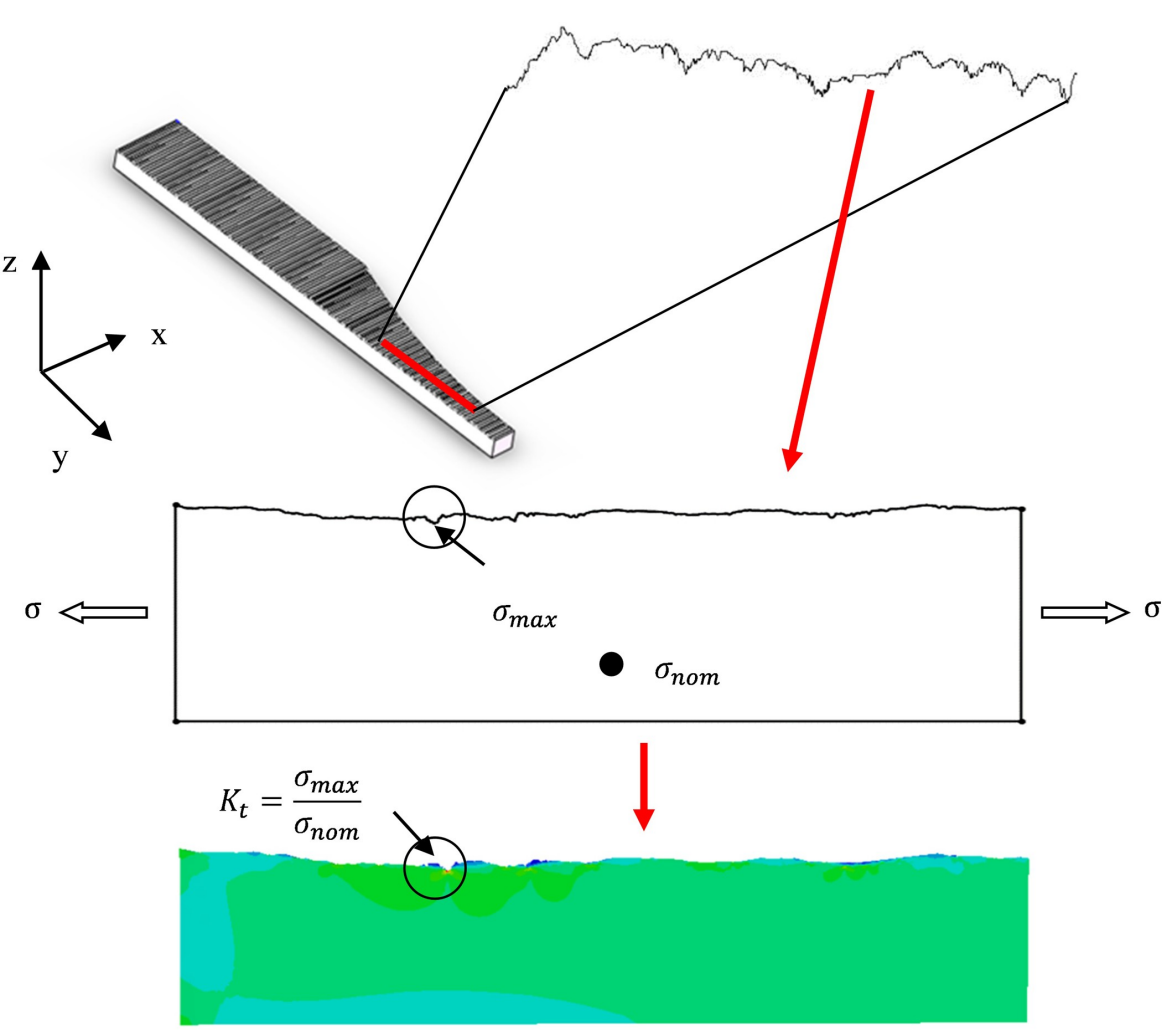
Principle of FE calculation to determine stress concentration factor.

Uniform load is applied as boundary conditions. The maximal Von Mises equivalent stress obtained by the calculation is then divided by the nominal Von Mises equivalent stress due to the applied load to classically determine the stress concentration factor *K*_*t*_ [14]. The processes of finite element calculation performed to determine *K*_*t*_ is shown in Fig 6.

### 2.6 Fatigue life estimation and validation

**Fig 7:**
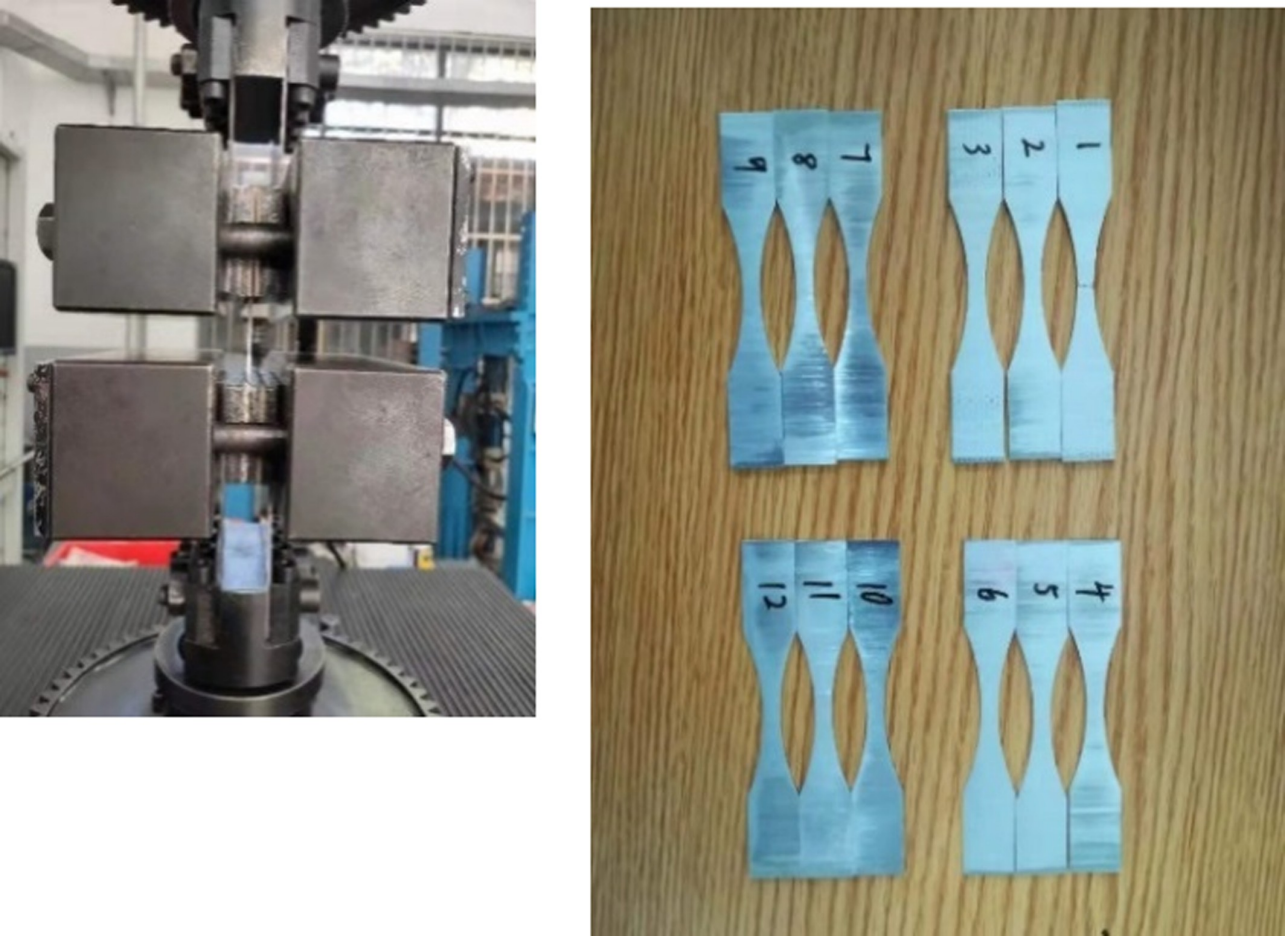
Fatigue test.

Fatigue testing is carried out to verify the predicted fatigue life from stress concentration factor. Fatigue testing is done under load control with load ratio *R* = 0.1, a frequency of 56 Hz and load value *F*_*max*_ = 3100N, by MTS testing system shown in Fig 7.

## 3 Results and discussion

### 3.1 Influence of surface micro-notch characteristics on stress concentration factor

#### 3.1.1 Stress concentration factor for single micro-notch condition

Theoretical stress concentration factor *K*_*t*_ = *σ*/*σ*_*nom*_ is introduced in this paper, where *σ* is the stress of notch root and *σ*_*nom*_ is the nominal stress of the section. The influence of single micro-notch parameters *a*,*b*,*a*/*b* on stress concentration factor is considered firstly.

**Fig 8:**
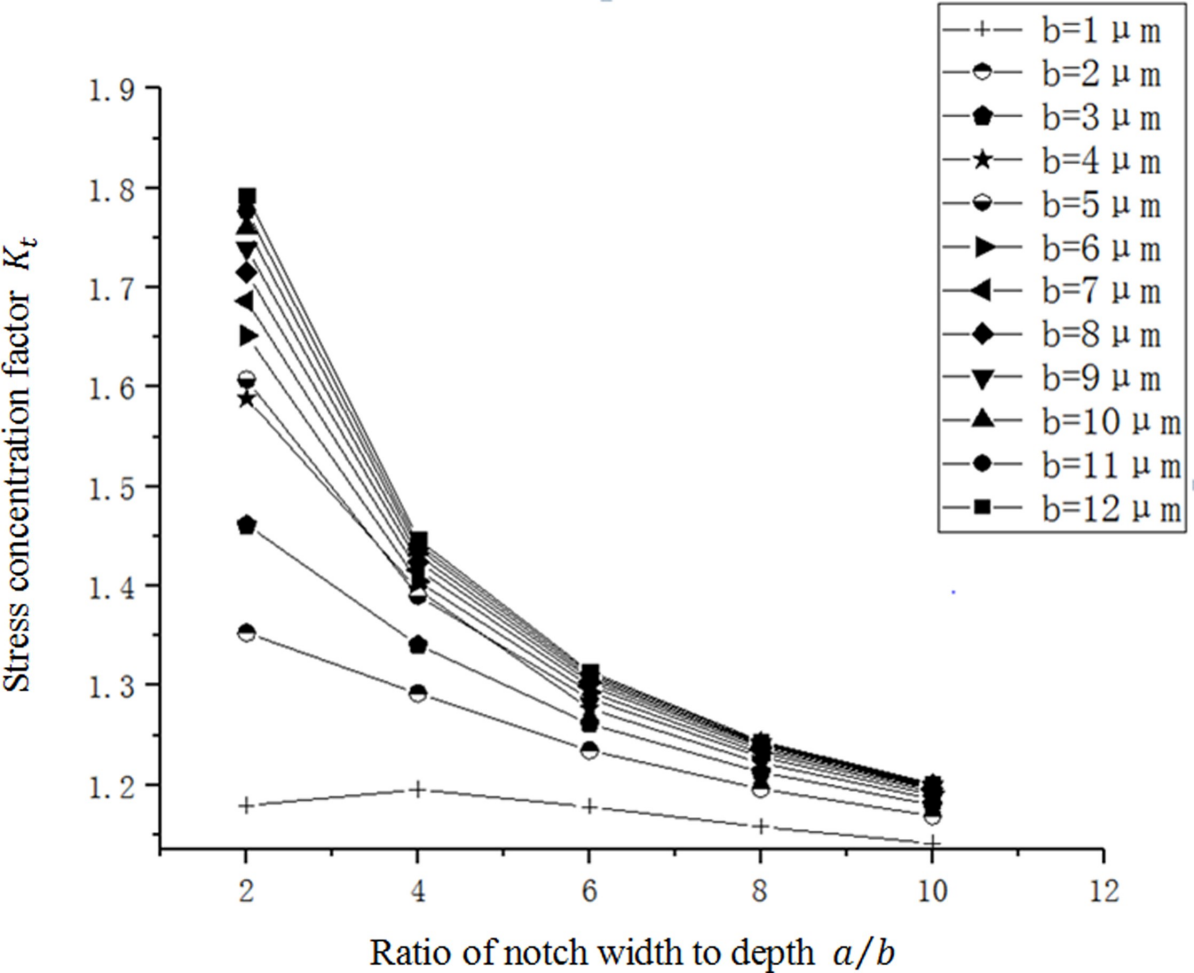
The relation curve between *K*_*t*_ and *a*/*b* in a single micro-notch.

As shown in Fig 8, the stress concentration factor *K*_*t*_ decreases with the increase of *a*/*b* gradually. *K*_*t*_ is not sensitive to the change of *a*/*b*, especially when a/b value is high. With the space between adjacent curves gradually decreasing, the increase of *a*/*b* value has gradually weakened the influence on *K*_*t*_. And *K*_*t*_ decreaseobservably when a/b is low, with largest influence between *a*/*b* equals 2 and 4. When the depth of notch *b* is relatively small, the curve changes gently, indicating that *K*_*t*_ is not sensitive to the change of *a*/*b* in this case. However, as the depth of notch *b* increases, the curve becomes steep and the value of *K*_*t*_ also increases.

**Fig 9:**
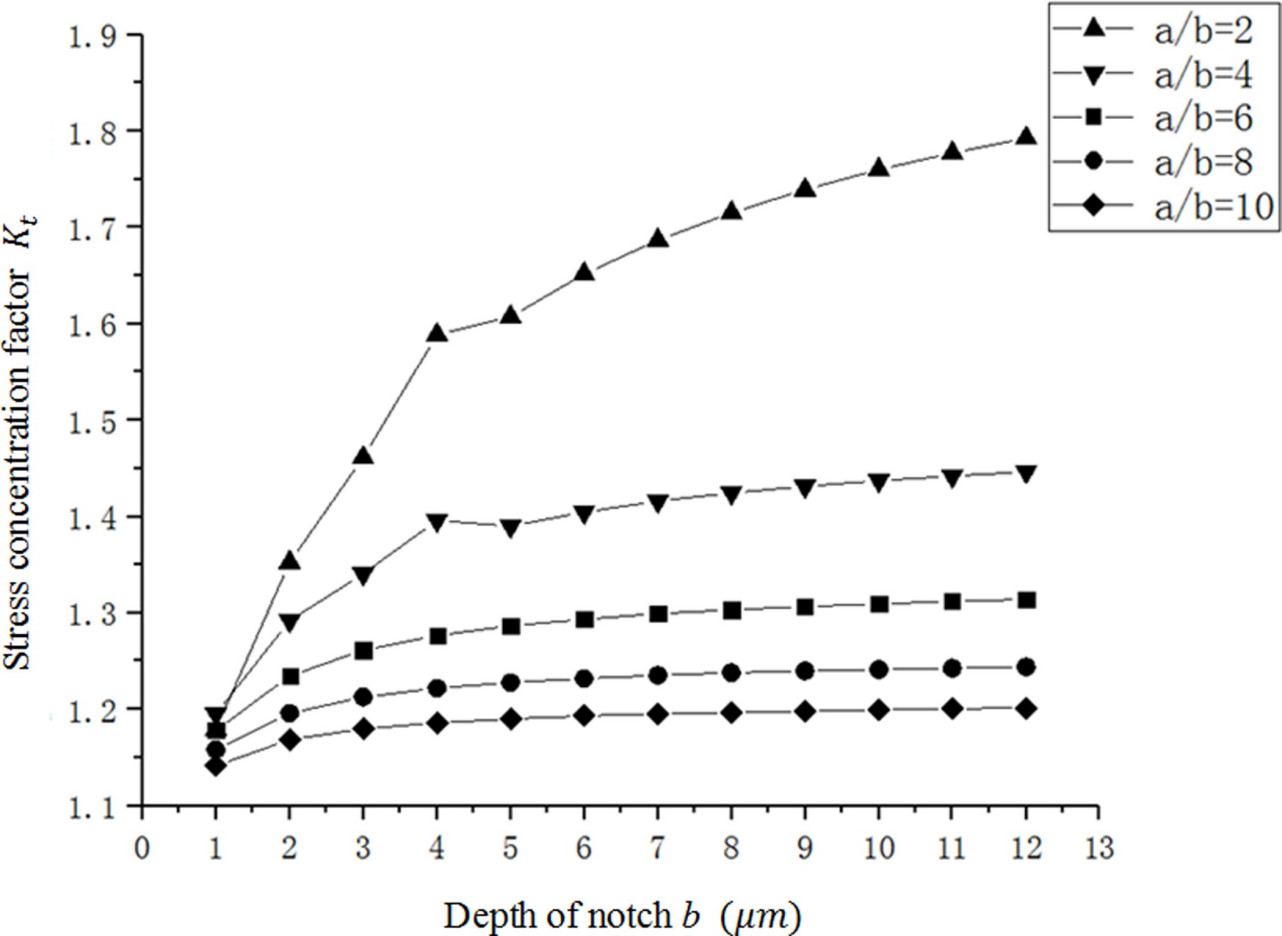
The relation curve between *K*_*t*_ and *b* in a single micro-notch.

The simulation data is fitted by polynomial fitting method in Fig 9, and an empirical formula of *K*_*t*_ is obtained for a single micro-notch with a correlation factor of 0.998 and a maximum error of 0.85%:

$$\begin{array}{l} K_{t}=0.93+0.37b-0.04{\text{b}}^{2}-0.07b(a/b)+0.02{\left(a/b\right)}^{2}+0.002b^{3}\\ +0.004b^{2}(a/b)+0.006b{\left(a/b\right)}^{2}-0.003{\left(a/b\right)}^{3} \end{array}$$ (1)

In order to verify the above empirical formula, several groups are randomly selected as notch parameters for simulation analysis, and the simulation results are compared with the fitting results, as shown in Table 1. The maximum error between the fitting value and the simulation value is 1.41%, indicating that the established empirical formula is reasonable.

**Table 1:** The fitting error of single micro-notch empirical formula.

*a/b*	*b*(μm)	Formula fitting value	Finite element simulation results	Error (%)
2.5	1.6	1.277	1.295	1.41%
2.5	6.3	1.589	1.576	-0.85%
5	1.6	1.234	1.237	0.24%
5	6.3	1.346	1.347	0.07%

#### 3.1.2 Stress concentration factor for multi-micro-notch condition

The primary notch parameters include center distance *d* of the notch, depth of notch *b* and ratio of notch width to depth *a*/*b*, and number of the notch *n*. Depth of the micro-notch *b* is 4μm, ratio of width to depth *a*/*b* is 2. The center distance of the notch *d* is 1~5 times the width of the notch 2*a*. And 1–21 continuous micro-notches are located symmetrically in the center of the model. Finite element model is established accordingly to calculate the multi-group *K*_*t*_ values. And the influence of the number of notches *n* on the stress concentration coefficient *K*_*t*_ is discussed.

**Fig 10:**
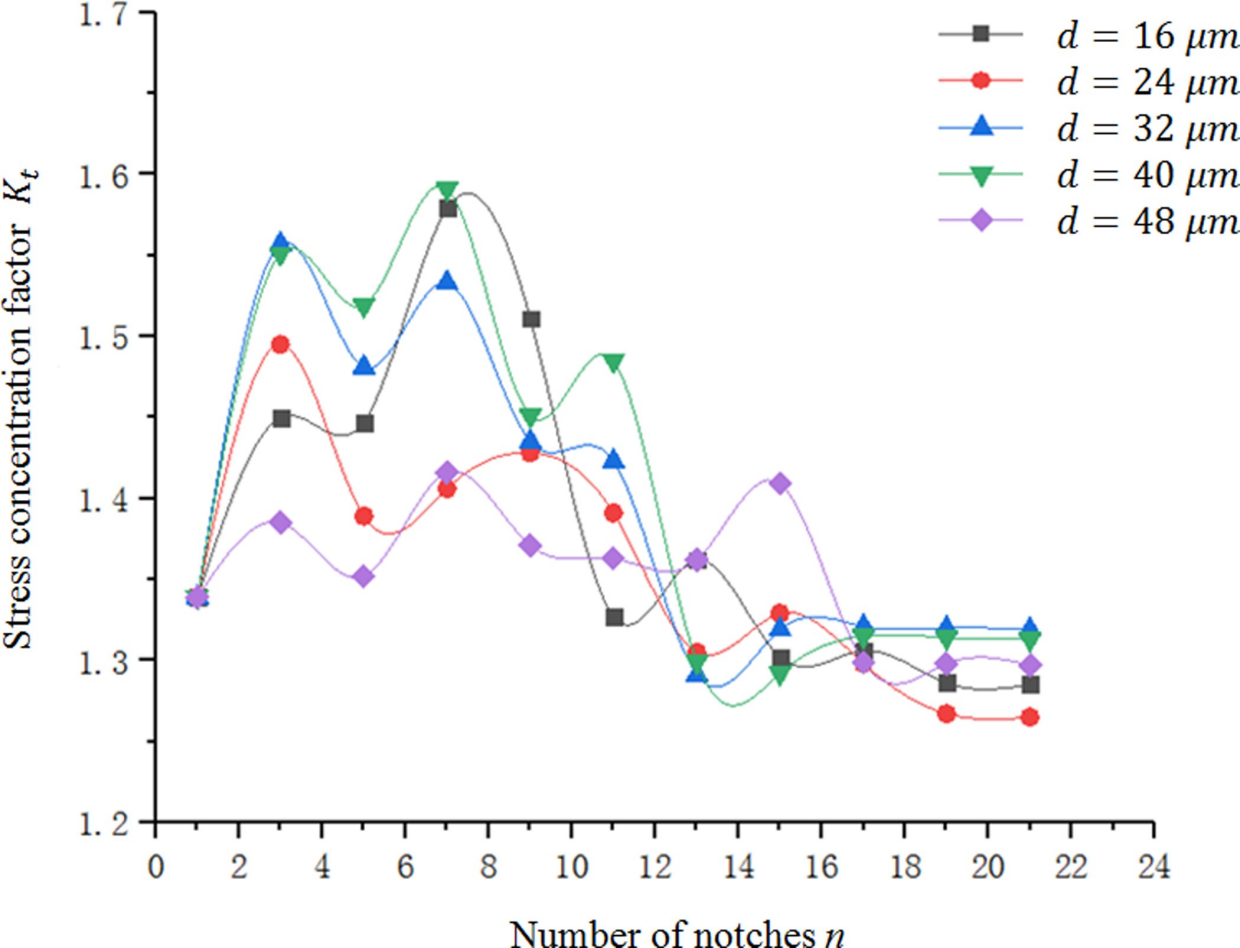
Relation between *n* and *K*_*t*_.

As shown in Fig 10, *K*_*t*_ first increases and then decreases with the increase of *n*, and the decreasing trend gradually becomes stable when *n* ≥ 19, and the value of *K*_*t*_ is consistent between the adjacent notches. Therefore, a sufficient number of micro-notches should be ensured to simulate the relevant characteristics of surface roughness and obtain relatively accurate quantitative relationship. In this paper, 21 identical equidistant notches are selected, and it is considered that the *K*_*t*_ value calculated by *n* = 21, when *n* = ∞.

**Fig 11:**
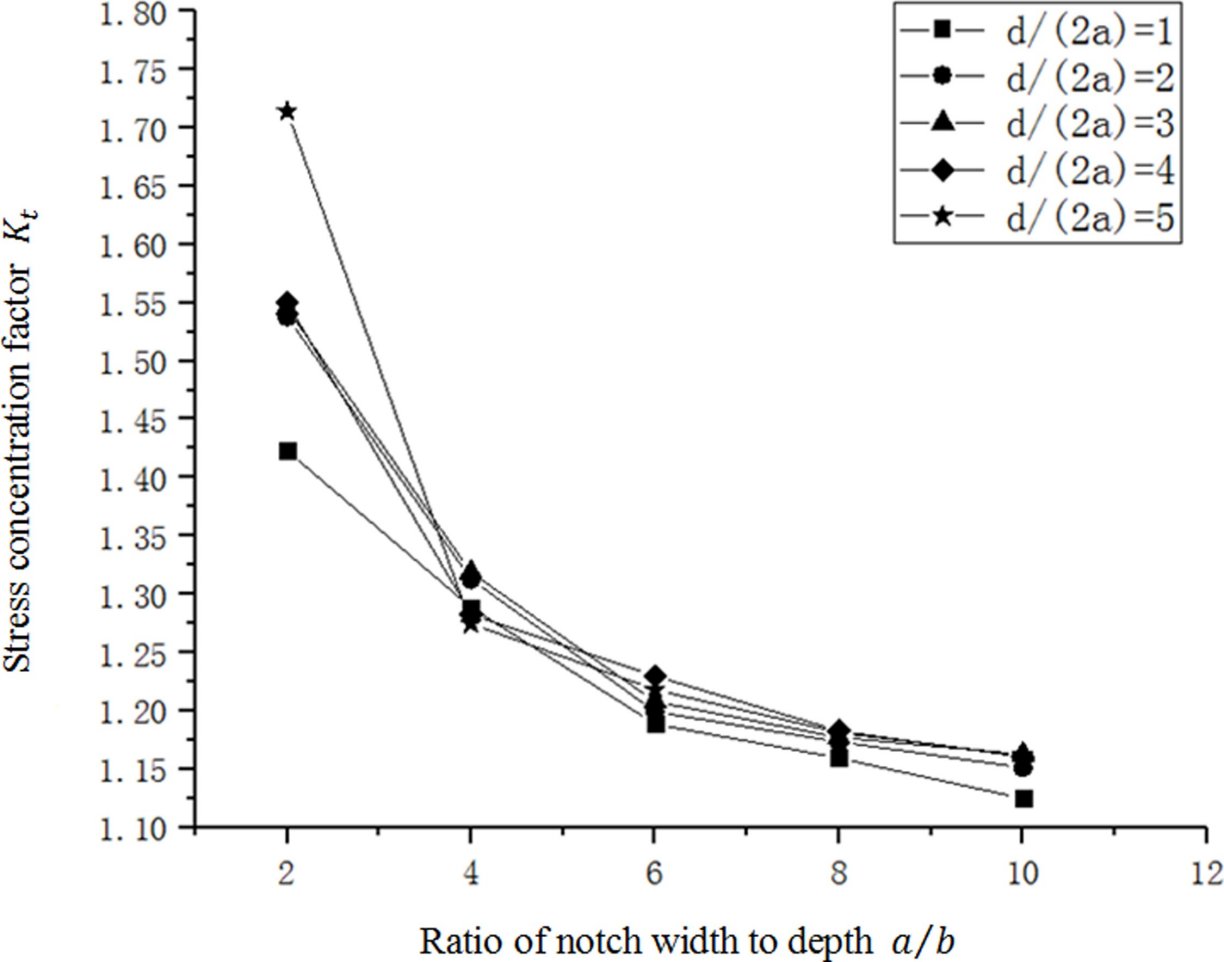
Relation between *a*/*b* and *K*_*t*_ of multi-micro-notches.

**Fig 12:**
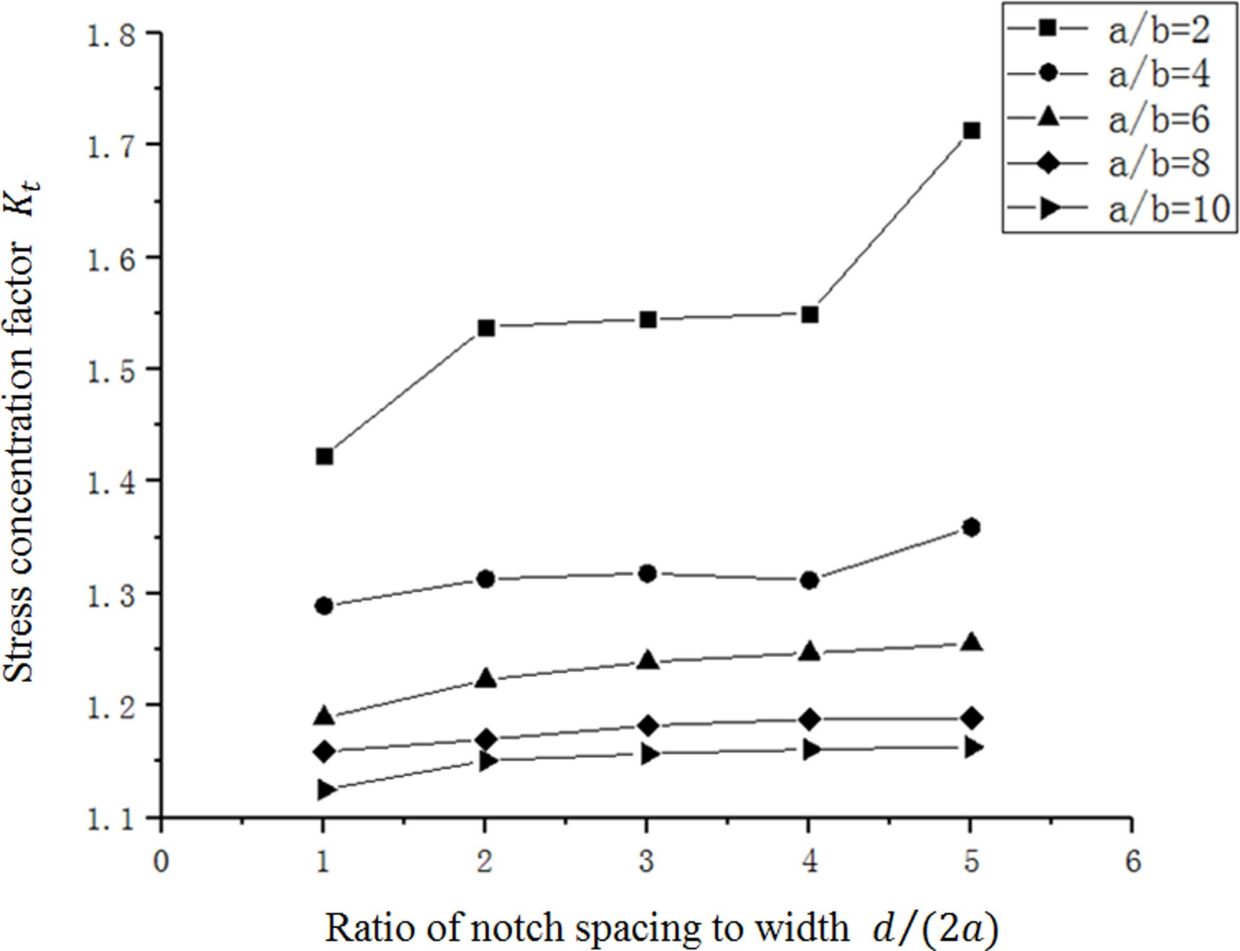
Relation between *d*/(2*a*) and *K*_*t*_ of multi-micro-notch.

As shown in Fig 11, the stress concentration factor gradually decreases as *a*/*b* increases. And in Fig 12, the stress concentration factor increases gradually, along with the increase of *d*/(2*a*). Comparing the curve of the single micro-notch curve with *b* = 4*μm*, it can be found that when multiple gaps are present, the stress concentration factor can be reduced. When *d*/(2*a*) = 5, the relation curve is very close between multi-micro notches and single-micro notch, which indicates that stress concentration can be alleviated only if *d* is less than a certain range. If the value of *d* is too large, the intersection between the gaps will be small, and the effect of reducing stress concentration is lost.

An empirical formula of *K*_*t*_ is obtained by the polynomial fitting for multi-micro-notches with a correlation coefficient of 0.989 and a maximum error of 1.63%:

$$\begin{array}{l} K_{t}=1.504+0.478(d/2a)-0.250(a/b)-0.130{\left(\text{d}/2a\right)}^{2}-0.092(d/2a)(a/b)\\ +0.061{\left(a/b\right)}^{2}+0.010{\left(d/2a\right)}^{3}+0.017{\left(d/2a\right)}^{2}\left(a/b\right) \end{array}$$ (2)

To verify this empirical formula, similar method is undertaken as the single notch analysis, by comparing the fitting and the simulation values, shown in Table 2. The maximum error between the fitting value and simulation values of stress concentration factor is -4.35%, which is acceptable as engineering error, indicating that the established empirical formula is reasonable and accurate.

**Table 2:** The fitting error of empirical formula for multi-micro-notches.

*a/b*	*d*/(2*a*)	Formula fitting value	Finite element simulation results	Error (%)
2	2.5	1.538	1.56	1.43
2	4.5	1.614	1.60	-0.88
2.5	2	1.455	1.417	-2.61
2.5	5	1.588	1.519	-4.35
4	1.5	1.308	1.289	-1.45
4	3.5	1.308	1.337	2.22

### 3.2 Empirical relation between surface roughness and stress concentration factor

**Fig 13:**
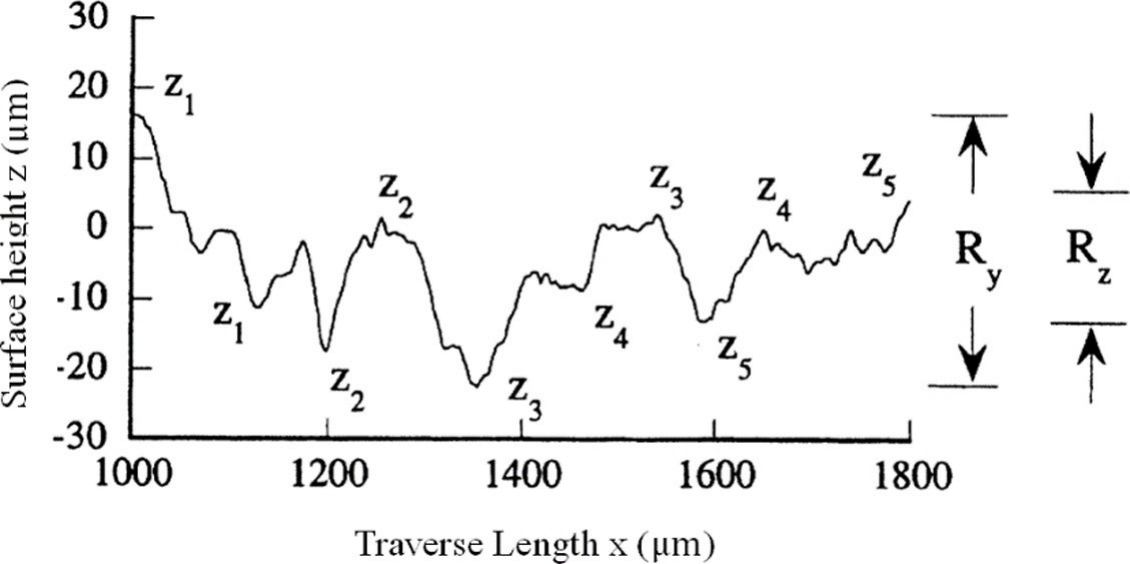
Arbitrary surface profile and standard surface roughness parameters.

A schematic description of those parameters for an arbitrary surface is shown in Fig 13 [19].

Note that *R*_*a*_ describes the average deviation in surface height from the profile mean line and *R*_*y*_ represents the height from the maximum peak to the lowest valley. *R*_*z*_ is a quantized value of the average height from the five highest peaks and five lowest valleys of the surface. Contour average unimodal spacing *S* refers to the distance along the midline between the highest points of two adjacent unimodal peaks. The mean interval *R*_*sm*_ of peak and valley profiles refers to the mean width *X*_*s*_ of all contour elements, and a continuous pair of peaks and valleys is called contour elements. The mathematical expression is shown as following:

$$R_{a}=\frac{1}{L}\int_{0}^{L}|z|\;dx$$ (3)

$$R_{y}=\left|z_{max}-z_{min}\right|$$ (4)

$$R_{z}=\frac{1}{5}\left[\sum_{i=1}^{5}{\left(z_{i}\right)}_{max}+\sum_{j=1}^{5}\left|{\left(z_{j}\right)}_{min}\right|\right]$$ (5)

$$\text{S}=\frac{1}{N}\sum_{1}^{N}S_{i}$$ (6)

$$R_{sm}=\frac{1}{n}\sum_{i=1}^{N}XS_{i}$$ (7)

Notch parameters *a*,*b* and notch spacing *d* can be converted by the 10-point roughness parameter *R*_*z*_ of standard surface roughness parameter, the single-peak average distance *S* of contour and the average interval *R*_*sm*_ of peak and valley profiles. It can be seen from the above mathematical expressions that the surface roughness parameters such as *R*_*z*_, *S* and *R*_*sm*_ are average values within a sampling length, while adjacent micro-notches in the simplified model is uniform distributed and the gap size is the same, which can also be considered as the average of a sample length, so they can be equivalent to *b* = *R*_*z*_/2, *d* = *S*, and *R*_*sm*_/2 = 2*a*. By substituting the above equivalent relation into Eq (2), relation between surface roughness and stress concentration factor is obtained:

$$\begin{array}{l} K_{t}=1.504+0.478(2S/R_{s}{}_{m})-0.250(R_{s}{}_{m}/(2R_{z})-0.130{\left(2\text{S}/R_{s}{}_{m}\right)}^{2}\\ -0.092(2S/R_{s}{}_{m})(R_{s}{}_{m}/(2R_{z})+0.061{(R_{s}{}_{m}/(2R_{z})}^{2}+0.010{\left(2S/R_{s}{}_{m}\right)}^{3}\\ +0.017{\left(2S/R_{s}{}_{m}\right)}^{2}(R_{s}{}_{m}/(2R_{z}) \end{array}$$ (8)

Surface roughness parameter values of *R*_*z*_, *R*_*sm*_ and *S* for the polished specimens are measured by surface roughness gauge, and substituted into Eq (8) to obtain the corresponding stress concentration factor value. Therefore, analysis time and cost can be reduced, comparing with traditional experimental methods.

### 3.3 Validation of roughness experiment

#### 3.3.1 Surface roughness testing

The roughness of artificial surface is measured longitudinally by contact method, shown in Table 3. The average roughness *R*_*a*_ ranges from 0.608 to 4.0μm, and 10-point roughness parameter *R*_*z*_ ranges from 5.647 to 23.844μm. From the first group to the fourth group, measured values of surface roughness parameters gradually decrease, and the surface become smoother.

**Table 3:** Roughness values of each specimen.

Specimen grouping	Specimen number	*R*_*a*_/μm	*R*_*z*_/μm	*R*_*sm*_/μm	*S*/μm
Group 1	1	3.468	21.567	66.83	61.43
	2	4.0	23.844	72.97	61.43
	3	3.125	20.846	95.23	71.73
Group 2	6	2.717	15.358	52.47	40.1
	7	2.561	15.102	52.77	38.93
	8	2.187	15.545	63.3	46.27
Group 3	9	1.780	11.025	38.13	28.97
	10	1728	12.996	41.43	30.23
	11	1.395	10.077	39	32.67
Group 4	12	0.737	5.921	27.1	27
	13	0.626	5.866	25.93	24.93
	14	0.608	5.647	25.03	22.2

By substituting the measured values into Eq (8), the fitted values of stress concentration factor corresponding to each group of test pieces can be obtained.

#### 3.3.2 Acquisition of three-dimensional shape feature of specimens

**Fig 14:**
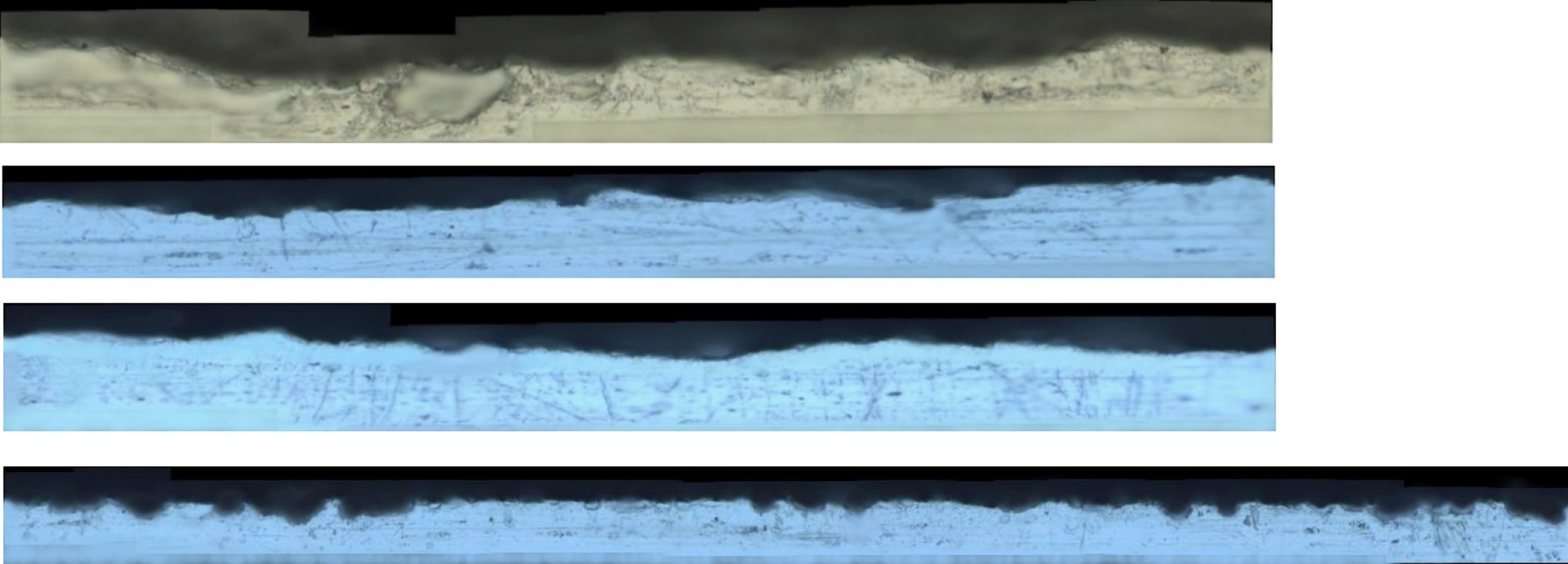
Picture of specimen surface topography.

The complete surface morphology of specimens obtained after splicing is shown in Fig 14.

**Fig 15:**
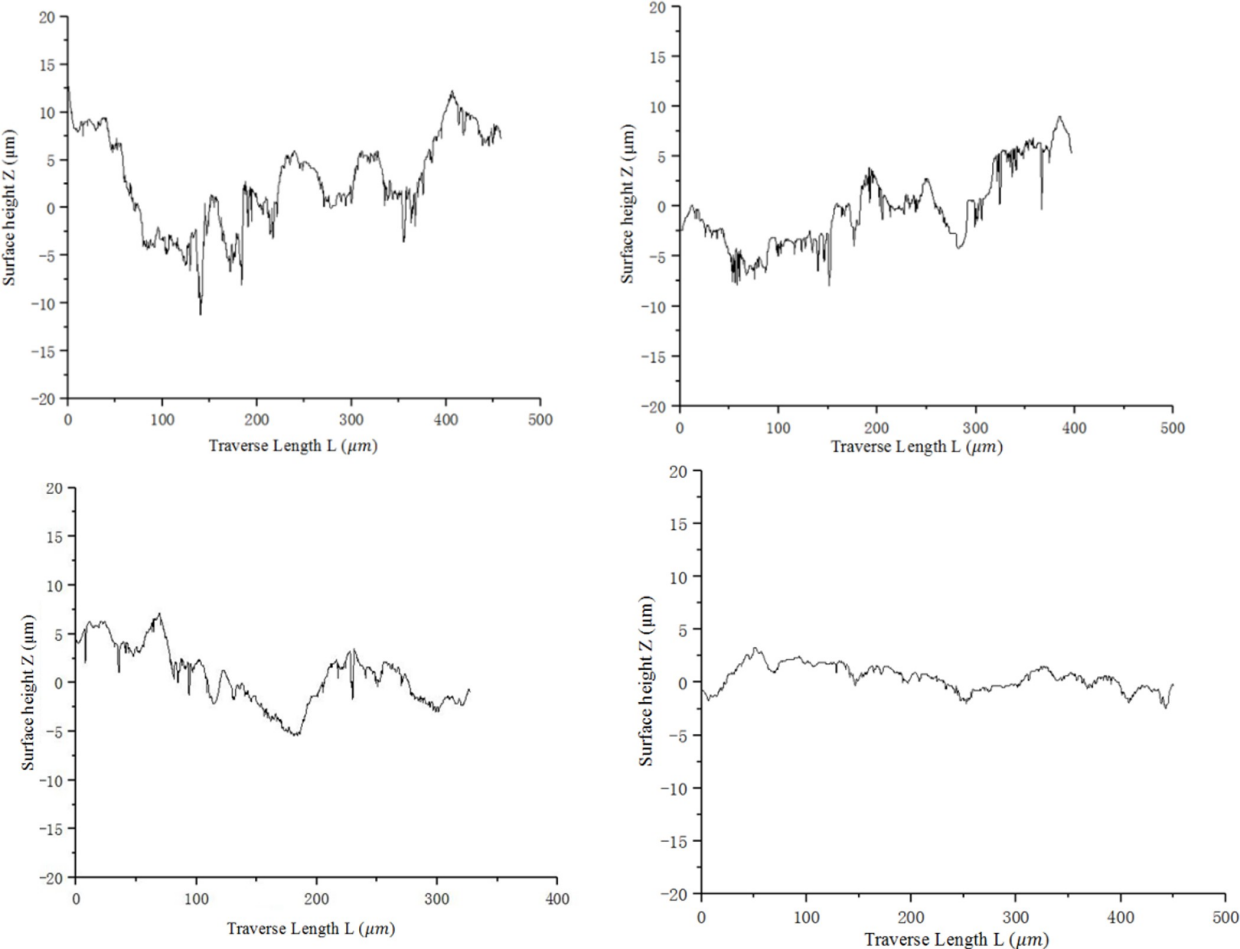
Surface morphology of specimens after grinding.

Fig 15 is the surface morphology of the four groups of 6061 aluminum alloy fatigue specimen. The surface dents of sandpaper after grinding are scattered, and when the dents are reduced, they overlap irregularly. The surface of the specimen after grinding with No. 80 sandpaper is relatively rough. The value of *R*_*z*_ for four groups fluctuate in the range of 5.759~23.962μm.

Comparing with real surface roughness measurement, certain deviation is observed for surface morphology method, with maximum value of 9.5%, shown in Table 4. One of the possible reasons may be the sampling position of the two when measuring, which is caused mainly due to the grinding process deviation. And another reason is possibly related to the accuracy deviation of the microscope. It is indicated that the real surface roughness of the test piece can be effectively represent by this contour curve extraction method. Through the calibration process, the true size of the acquired image can be obtained, and positioning is performed for the subsequent extraction of the boundary curve. Then, the surface topography data points after interpolation are imported into 3D modeling software through point cloud technology to generate curve to finish surface reconstruction.

**Table 4:** Maximum dent depth value *R*_*z*_ of specimens observed by microscope.

Specimen grouping	Specimen number	*R*_*z*_/μm	*R*_*z*_ average	Error (%)
Group1	1	23.962	21.682	1.82
	2	22.661		
	3	18.424		
Group 2	6	16.264	16.736	-9.14
	7	17.316		
	8	16.627		
Group 3	9	12.359	12.446	-9.5
	10	12.473		
	11	12.505		
Group 4	12	5.763	5.783	0.48
	13	5.827		
	14	5.759		

#### 3.3.3 FE calculation of *K*_*t*_

Base on the above model, finite element model is established, using quarter-symmetric model. The processes of finite element calculation performed to determine *K*_*t*_ is shown in Fig 6. It is shown that *K*_*t*_ obtained by finite element analysis of measured topography of surface fits well with the simplified model (Table 5). The maximum error is 1.25%, which indicates the correctness and rationality of the empirical relationship between surface roughness and stress concentration coefficient.

**Table 5:** Empirical formula fitting error for *K*_*t*_.

Specimen grouping	Specimen number	Simulation value *K*_*t*_	Fitted value *K*_*t*_	Error (%)
Group 1	1	1.6	1.618	1.13
	2	1.608	1.624	0.99
	3	1.601	1.615	0.87
Group 2	6	1.534	1.547	0.85
	7	1.542	1.558	1.04
	8	1.535	1.551	1.04
Group 3	9	1.52	1.529	0.59
	10	1.515	1.534	1.25
	11	1.51	1.522	0.79
Group 4	12	1.465	1.471	0.41
	13	1.462	1.47	0.55
	14	1.455	1.461	0.41

## 4 Analysis of fatigue life

### 4.1 Theoretical estimation

Combined with the comprehensive expression of fatigue crack initiation life given in [20], fatigue life under different surface roughness can be analyzed. The formula given is as follows:

$$N_{i}=C{\left[\Delta \sigma_{e}{{}_{qv}}^{2/(1+n)}-{\left(\Delta_{e}{}_{qv}\right)}_{t}{{}_{h}}^{2/(1+n)}\right]}^{-2}$$ (9)

where *C* is crack initiation resistance coefficient, which is a material constant related to tensile properties, *n* is strain hardening exponents. (Δ*σ*_*eqv*_)_*th*_ is threshold for initiation of cracks expressed in equivalent stress amplitude, which is related to tensile properties and fatigue limit material constant, and (Δ*σ*_*eqv*_ is equivalent stress amplitude, which can be expressed as:

$$\Delta \sigma_{eqv}=\sqrt{\frac{1}{2(1-R)}}K_{t}\cdot \Delta S$$ (10)

where *K*_*t*_ is theoretical stress concentration factor, *R* is stress ratio (*R* = *S*_*min*_/*S*_*max*_), and Δ*S* is nominal stress range (ΔS = *S*_*max*_/*S*_*min*_).

According to the fatigue test conditions, the estimated performance parameter values of 6061-T6 aluminum alloy are shown in Table 6.

**Table 6:** Estimated performance parameter values (MPa).

Materials	*E*	*n*	*S*_*max*_	*S*_*min*_	Δ*σ*_*eqv*_	*C*
6061-T6	68900	0.085	309	30.9	127	3.53×10^13^

Based on the above conditions, the fatigue crack initiation formula of 6061-T6 aluminum alloy can be obtained:

$$N_{i}=3.53\times 10^{1}{}^{3}{\left[{\left(207.28K_{t}\right)}^{1}{}^{.84}-127^{1}{}^{.84}\right]}^{-2}$$ (11)

### 4.2 Validation

The prediction of fatigue life by substituting the fitted value of the stress concentration factor into the formula (11) is compared with fatigue testing data. Table 7 is the comparison of fatigue life for both model prediction and experiment testing.

**Table 7:** Comparison of fatigue life for prediction and experiment.

Specimen grouping	Specimen number	Prediction life	Experiment life	Error (%)
Group 1	1	25884	29426	-12.03
	3	26097	26621	-1.97
Group 2	6	31617	33639	-6.01
	8	31252	29124	7.31
Group 3	9	33330	39514	-15.65
	11	34029	32406	5
Group 4	12	39755	41687	-4.63
	14	41024	36329	12.92

Two specimens of each group have been selected for the comparison. It is found that the fatigue life predicted through the established relationship is in good agreement with the test results overall, with a maximum error of 15.65%. The reasons of the errors are not only the mesh refinement around notches, but also the deviation of the grinding for the surface roughness. At the same time, the low cycle fatigue of test may yield plasticity to relieve fatigue, that is the reason the test values are larger generally. However, the overall trend was consistent with each other. It is illustrated that the surface roughness has a certain influence on the fatigue life of specimens, and the larger the surface roughness, the smaller the fatigue life. It is further explained that the surface roughness can be simplified into a notch model and the corresponding relationship can be utilized for fatigue life prediction.

## 5 Conclusions

In this paper, the surface roughness is simplified into a series of elliptical micro-notches, and the empirical relationship between surface roughness and fatigue life is established through FE simulation analysis. At the same time, a method for obtaining real surface roughness contour curve based on image processing is proposed, and the 3D model reconstruction based on the actual surface roughness is completed. The empirical relationship established is compared and verified through FE analysis and experiment. The following conclusions can be drawn.

1. An empirical relationship between the micro-notch parameters and the stress concentration factor is established. Surface roughness parameters are selected to represent the micro notch parameters according to the mathematical definition, such as *R*_*z*_, S, *R*_*sm*_. And the relationship between the surface roughness parameters and the stress concentration factor is established.
2. The proposed extraction method of surface curve can effectively represent the true surface roughness of the test piece. Comparing the results between simulation and calculation, it is found that the maximum error is only 1.25%, indicating that the simplified model of the gap can well reflect the true stress concentration of the test specimen.
3. The relationship between the surface roughness parameter and the fatigue life is established, and the predicted value of the fatigue life is compared with the test results. It is illustrated that the change trend of the two is basically the same, and the maximum error is 15.65%, indicating that the formula established is reasonable and effective.

## Supporting information

S1 File (PDF)
