# Supplementary material for: Fatigue life analysis for 6061-T6 aluminum alloy based on surface roughness
Source: PLoS One. 2021 Jun 30;16(6):e0252772. doi: 10.1371/journal.pone.0252772 (PMC8244873; doi:10.1371/journal.pone.0252772)
Supplement: S1 File — (PDF) [file pone.0252772.s001.pdf]

S1_Table 1. The fitting error of single micro-notch empirical formula

a/b	b	**Result**
2.5	1.6	194.25
2.5	6.3	236.4
5	1.6	185.55
5	6.3	202.05

a/b	b	**Result**
2.5	1.6	1.295
2.5	6.3	1.576
5	1.6	1.237
5	6.3	1.347

S2_Table 2. The fitting error of empirical formula for multi-micro-notches

a/b	d/(2a)	**Result**
2	2.5	234
2	4.5	240
2.5	2	212.55
2.5	5	227.85
4	1.5	193.35
4	3.5	200.55

a/b	d/(2a)	**Result**
2	2.5	1.56
2	4.5	1.6
2.5	2	1.417
2.5	5	1.519
4	1.5	1.289
4	3.5	1.337

S5_Table 5. Empirical formula fitting error for Kt

80-2	1.6
80-3	1.608
80-4	1.601
120-2	1.534
120-3	1.542
120-4	1.535
240-2	1.52
240-3	1.515
240-4	1.51
400-2	1.465
400-3	1.462
400-4	1.455

S8_Figure 8. The relation curve between K_t and a/b in a single micro-notch

b	2	4	6	8	10
1	176.9	179.3	176.7	173.7	171.2
2	202.9	193.8	185.2	179.4	175.3
3	219.2	201.1	189.2	181.9	177
4	238.3	209.4	191.5	183.3	177.9
5	241.1	208.5	193	184.2	178.5
6	247.8	210.7	194	184.8	179
7	253	212.4	194.9	185.3	179.3
8	257.3	213.7	195.5	185.7	179.5
9	260.9	214.7	196	186	179.7
10	264	215.6	196.4	186.2	179.9
11	266.6	216.3	196.8	186.4	180.1
12	268.9	217	197.1	186.6	180.2

Unit：Mpa

b	2	4	6	8	10
1	1.179333	1.195333	1.178	1.158	1.141333
2	1.352667	1.292	1.234667	1.196	1.168667
3	1.461333	1.340667	1.261333	1.212667	1.18
4	1.588667	1.396	1.276667	1.222	1.186
5	1.607333	1.39	1.286667	1.228	1.19
6	1.652	1.404667	1.293333	1.232	1.193333
7	1.686667	1.416	1.299333	1.235333	1.195333
8	1.715333	1.424667	1.303333	1.238	1.196667
9	1.739333	1.431333	1.306667	1.24	1.198
10	1.76	1.437333	1.309333	1.241333	1.199333
11	1.777333	1.442	1.312	1.242667	1.200667
12	1.792667	1.446667	1.314	1.244	1.201333

S9_Figure 9.The relation curve between K_t and b in a single micro-notch　　　　　　Unit：Mpa

a	1	2	3	4	5	6	7	8	9	10	11	12
2	176.9	202.9	219.2	238.3	241.1	247.8	253	257.3	260.9	264	266.6	268.9
4	179.3	193.8	201.1	209.4	208.5	210.7	212.4	213.7	214.7	215.6	216.3	217
6	176.7	185.2	189.2	191.5	193	194	194.9	195.5	196	196.4	196.8	197.1
8	173.7	179.4	181.9	183.3	184.2	184.8	185.3	185.7	186	186.2	186.4	186.6
10	171.2	175.3	177	177.9	178.5	179	179.3	179.5	179.7	179.9	180.1	180.2

a	1	2	3	4	5	6	7	8	9	10	11	12
2	1.179333	1.352667	1.461333	1.588667	1.607333	1.652	1.686667	1.715333	1.739333	1.76	1.777333	1.792667
4	1.195333	1.292	1.340667	1.396	1.39	1.404667	1.416	1.424667	1.431333	1.437333	1.442	1.446667
6	1.178	1.234667	1.261333	1.276667	1.286667	1.293333	1.299333	1.303333	1.306667	1.309333	1.312	1.314
8	1.158	1.196	1.212667	1.222	1.228	1.232	1.235333	1.238	1.24	1.241333	1.242667	1.244
10	1.141333	1.168667	1.18	1.186	1.19	1.193333	1.195333	1.196667	1.198	1.199333	1.200667	1.201333

S10_Figure 10. Relation between n and K_t **Unit：Mpa**

n \ d	16	24	32	40	48
1	200.8	200.8	200.8	200.8	200.8
3	217.5	224.3	233.6	232.7	207.7
5	217	208.4	222.1	227.8	202.8
7	236.9	210.9	229.9	238.7	212.4
9	226.6	214.2	215.3	217.7	205.7
11	199.1	208.6	213.4	222.8	204.4
13	204.3	195.7	193.6	194.8	204.3
15	195.3	199.3	197.8	193.8	211.3
17	195.9	194.7	198.2	197.3	194.8
19	192.9	190.1	198	197.1	194.7
21	197.8	189.8	197.8	197	194.6

n \ d	16	24	32	40	48
1	1.338667	1.338667	1.338667	1.338667	1.338667
3	1.45	1.495333	1.557333	1.551333	1.384667
5	1.446667	1.389333	1.480667	1.518667	1.352
7	1.579333	1.406	1.532667	1.591333	1.416
9	1.510667	1.428	1.435333	1.451333	1.371333
11	1.327333	1.390667	1.422667	1.485333	1.362667
13	1.362	1.304667	1.290667	1.298667	1.362
15	1.302	1.328667	1.318667	1.292	1.408667
17	1.306	1.298	1.321333	1.315333	1.298667
19	1.286	1.267333	1.32	1.314	1.298
21	1.318667	1.265333	1.318667	1.313333	1.297333

S11_Figure 11. Relation between a/b and K_t of multi-micro-notches

Unit：Mpa

a/b \ d/(2a)	1	2	3	4	5
2	213.45	230.7	231.75	232.5	257.1
4	193.35	196.95	197.7	196.8	203.85
6	178.35	183.45	185.85	187.05	188.25
8	173.85	175.5	177.3	178.2	178.35
10	168.75	172.65	173.55	174.15	174.45

a/b \ d/(2a)	1	2	3	4	5
2	1.423	1.538	1.545	1.55	1.714
4	1.289	1.313	1.318	1.312	1.359
6	1.189	1.223	1.239	1.247	1.255
8	1.159	1.17	1.182	1.188	1.189
10	1.125	1.151	1.157	1.161	1.163

S12_Figure 12 Relation between d(2a) and K_t of multi-micro-notch

a/b<br>d/(2a)	2	4	6	8	10
1	213.45	193.35	178.35	173.85	168.75
2	230.7	196.95	183.45	175.5	172.65
3	231.75	197.7	185.85	177.3	173.55
4	232.5	196.8	187.05	178.2	174.15
5	257.1	203.85	188.25	178.35	174.45

Unit：Mpa

a/b<br>d/(2a)	2	4	6	8	10
1	1.423	1.289	1.189	1.159	1.125
2	1.538	1.313	1.223	1.17	1.151
3	1.545	1.318	1.239	1.182	1.157
4	1.55	1.312	1.247	1.188	1.161
5	1.714	1.359	1.255	1.189	1.163
